# Case Report: A Spinal Ischemic Lesion in a 24-Year-Old Patient With Fabry Disease

**DOI:** 10.3389/fimmu.2020.595514

**Published:** 2020-12-14

**Authors:** Julia Krämer, Felix Glaser, Martin Hasselblatt, Eva Brand, Christian Pogoda, Malte Lenders, Heinz Wiendl, Sven G. Meuth, Thomas Duning

**Affiliations:** ^1^ Department of Neurology with Institute of Translational Neurology, University Hospital Münster, Münster, Germany; ^2^ Institute of Neuropathology, University Hospital Münster, Münster, Germany; ^3^ Department of Internal Medicine D and Interdisciplinary Fabry Center (IFAZ), University Hospital Münster, Münster, Germany; ^4^ Department of Cardiology I—Coronary and Peripheral Vascular Disease and Heart Failure and Interdisciplinary Fabry Center (IFAZ), University Hospital Münster, Münster, Germany; ^5^ Department of Neurology, University Hospital Düsseldorf, Düsseldorf, Germany

**Keywords:** Fabry disease, spinal lesion, multiple sclerosis, spinal ischemia, case report, MRI

## Abstract

**Background:**

While cerebral lesions are common in Fabry disease (FD), spinal lesions have not been described, and their presence was suggested to be indicative of multiple sclerosis. Here, we present a FD patient with histopathological confirmed spinal ischemic stroke.

**Case presentation:**

A patient with genetically and biochemically diagnosed FD and characteristic manifestations (acroparesthesia, angiokeratomas, hypohidrosis, microalbuminuria, myocardial hypertrophy) presented with paraplegia, loss of all sensory modalities below Th9, and loss of bowel and bladder function. While cranial MRI was inconspicuous, spinal MRI showed a T2 hyperintense, non-contrast-enhancing lesion of the thoracic spinal cord. Lumbar puncture revealed mild pleocytosis, increased total protein and lactate levels, decreased glucose ratio, and negative oligoclonal bands. Rheumatic, neoplastic, and infectious disorders were excluded. The patient received intravenous and intrathecal methylprednisolone, plasmapheresis, intravenous immunoglobulins, and cyclophosphamide without clinical improvement. A biopsy of the thoracic lesion was performed. A histopathological examination revealed necrotic tissue consistent with spinal cord ischemia. Diagnostic work-up for stroke etiology clarification was not conspicuous. Two years onward, the patient suffered from a pontine infarction and a transient ischemic attack.

**Conclusion:**

The current case highlights the possible occurrence of spinal ischemic lesions in FD. Thus, the diagnosis of FD should not be prematurely discarded in the presence of spinal lesions.

## Introduction

Fabry disease (FD) is a rare, multiorgan, life-threatening, X-linked inherited lysosomal storage disorder with central and peripheral nervous system involvement, including cerebral micro- and macroangiopathy and neuropathic pain (1, 2).

Cerebral MRI mainly shows a variable degree of non-specific distributed T2-weighted and fluid-attenuated inversion recovery (FLAIR) hyperintense white matter lesions (WMLs). Ischemic strokes frequently occur before diagnosis, and there is an inconstant appearance of dilative arteriopathy in the vertebrobasilar system (2–4).

FD is commonly misdiagnosed as multiple sclerosis (MS) because patients with either disease may present with sensory symptoms, pain, cognitive impairment, and WMLs. Although microangiopathic cerebral lesions are common, spinal lesions are not described in FD. Thus, some authors suggest that the absence of spinal lesions should prompt clinicians to revise an initial diagnosis of MS to FD (5). Here, we present a FD patient with histopathological confirmed spinal ischemic stroke.

## Case Report

### Patient Information

A 24-year-old male Caucasian was first diagnosed with FD in June 2010. FD was genetically and biochemically confirmed (hemizygote mutation of α-galactosidase A (α-Gal A), chromosome Xq22, exon 2 (IVS2+1G>T); **Table 1**). Until that day, he suffered from acroparesthesia triggered by physical stress and exogenous heat since childhood, hypohidrosis, and classical angiokeratomas of the umbilical and genital area. Urine diagnostics and transesophageal echocardiography were inconspicuous. The patient regularly received enzyme replacement therapy with agalsidase alfa since 2011, leading to a significant improvement of neuropathic symptoms. Symptomatic treatment consisted of pregabalin and duloxetine. In April 2014, microalbuminuria (albumin/creatinine ratio 218 mg/g) and mild concentric myocardial hypertrophy (interventricular septum thickness of 11 mm) were detected. Therapy with angiotensin receptor blockers was initiated.

**Table 1:** Biochemical findings and laboratory data of the Fabry disease (FD) patient.

Biochemical findings*	Result (reference value)
α-Gal A enzyme activity level	1 nmol/MU/mg (> 33 nmol/MU/mg)
globotriaosylsphingosine (lyso-Gb3) level	29 ng/ml (< 2.2 ng/ml)
Pathogen diagnostics in serum	negative
Human T-cell leukemia virus type 1 and 2 Tick-borne meningoencephalitis (FSME) Hepatitis B and C HIV Borrelia burgdorferi Treponema pallidum	negative negative negative negative negative negative
Pathogen diagnostics in CSF	negative
Herpes simplex virus type 1 and 2 Varicella-zoster virus Human herpesvirus 6 Enterovirus	negative negative negative negative
Borrelia burgdorferi FSME Treponema pallidum	negative negative negative
Antibody screening in serum	negative
Anti-neuronal antibodies Aquaporin-4 antibodies Myelin oligodendrocyte glycoprotein antibodies Anti-ganglioside antibodies	negative negative negative negative

*Before initiation of enzyme replacement therapy.

### Family History

The patient’s mother with genetically and biochemically confirmed FD also suffered from acroparesthesia triggered by physical stress and exogenous heat. She is also treated with agalsidase.

### Clinical Findings

In February 2016, the patient presented to our clinic with a complete spinal cord syndrome with paraplegia, loss of all sensory modalities below Th9, and loss of bowel and bladder function. The symptoms developed within one day.

### Diagnostic Assessment

**Figure 1:**
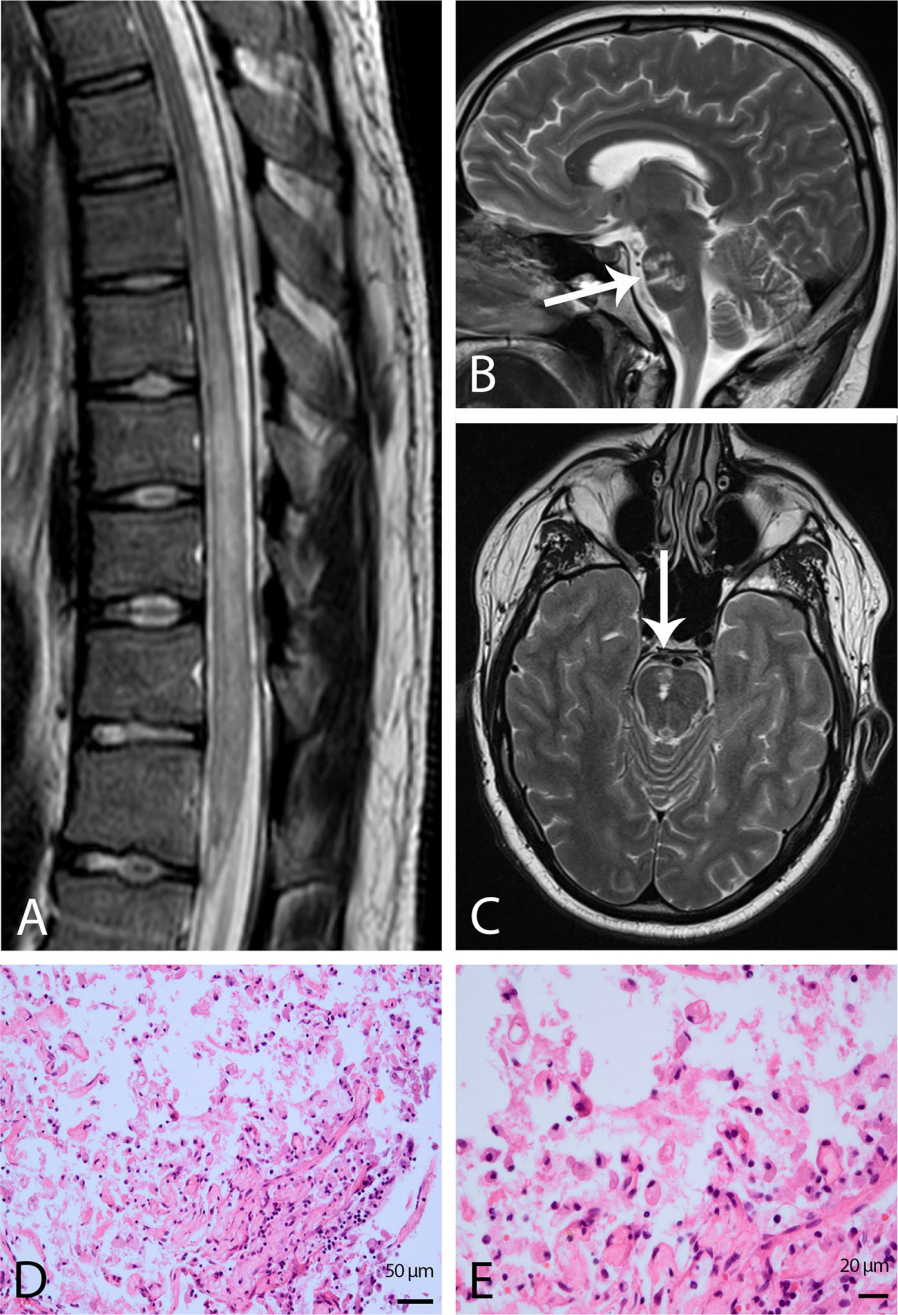
Findings from MRI and histological examination of spinal cord ischemia. **(A)** Sagittal T2-weighted image of the thoracic spinal cord demonstrating a pronounced hyperintensity from the seventh to the twelfth thoracic vertebra. Sagittal **(B)** and axial **(C)** T2-weighted turbo spin-echo images of the brain displaying right-sided pontine infarction which is marked by a white arrow. Hematoxylin and eosin (H&E) staining of myelon biopsy displaying necrotic central nervous system tissue with macrophages. Original magnification 20x **(D)** and 40x **(E)**.

Cranial MRI during hospitalization was unrevealing. Spinal MRI demonstrated a pronounced T2 hyperintense lesion of the thoracic spinal cord that did not enhance after gadolinium administration (**Figure 1A**). The diffusion-weighted imaging showed no signal loss in the apparent diffusion coefficient map. Lumbar puncture revealed a mild pleocytosis (19 lymphocytes/µl and 1 granulocyte/µl), elevated total protein (1,580 mg/L) and lactate levels (3.37 mmol/L; reference value 1.5–2.1 mmol/L), and decreased cerebrospinal fluid (CSF)/blood glucose ratio (0.43; reference value 0.6–0.9). Oligoclonal bands were negative. Infectious disorders were excluded as possible causes (**Table 1**). A detailed antibody screening (**Table 1**) was negative, as well as blood tests for vasculitis and autoimmune rheumatic systemic diseases.

### Treatment and Outcome

Under the suspicion of autoimmune disease, high-dose intravenous methylprednisolone (2 x 5 g), eight cycles of plasmapheresis, intrathecal methylprednisolone (120 mg), and five times intravenous immunoglobulins (150 g overall) were administered. Immunosuppressive therapy with cyclophosphamide was initiated. Due to a lack of improvement, the patient had a diagnostic re-evaluation one month later. Spinal MRI demonstrated unchanged findings, and whole-body ^18^F-FDG-PET/CT to exclude malignancies revealed no clarifying results. Repeated lumbar puncture showed persisting elevated total protein levels (1,040 mg/L) but no more pleocytosis and still negative oligoclonal bands. The patient received another eight cycles of plasmapheresis, which did not lead to clinical improvement. Finally, a biopsy of the thoracic lesion was performed by hemilaminectomy for further clarification. The histological examination revealed necrotic tissue consistent with previous spinal cord ischemia (**Figures 1D, E**). Therefore therapy with aspirin and statin was initiated. Extensive diagnostic work-up, including computed tomography of the thorax, long-term ECG, and transthoracic echocardiography, was carried out to clarify the stroke etiology but was unrevealing. Two years onward, the patient suffered from a pontine infarction (**Figures 1B, C**) with vertigo and weakness of the left arm, and one month later, a transient ischemic attack with latent right-sided hemiparesis occurred. Long-term ECG and transesophageal echocardiography were inconspicuous. The spinal cord syndrome persisted so that the patient remains wheelchair-bound and catheterized. The patient is regularly examined in our clinic.

## Discussion

WMLs are early and frequent findings in FD. Thus, FD was suggested as a model disease for cerebral microangiopathy (3). Cerebral small vessel involvement in FD patients was described as endothelial cell dysfunction due to the deposition of neutral glycosphingolipids (6). The pattern of WMLs frequently referred to as “vascular leukodystrophy” may vary in appearance due to aging and cerebral lesion load and can confound the differential diagnosis (7). MS has been proposed as a differential diagnosis for FD. FD patients are commonly misdiagnosed with MS due to cerebral lesions, sensory symptoms, pain, and similar age of manifestation. Any spinal cord involvement with characteristic neuroradiological findings was suggested as a powerful diagnostic pointer toward MS. Although cerebral WMLs are common, spinal lesions are not described in patients with FD and are even used to distinguish between MS and FD (5). To date, spinal MRI lesions were described only in one patient with FD who was diagnosed with a coexistent inflammatory demyelinating disease (8).

Our case is the first report of spinal ischemic stroke in a patient with Fabry-typical clinical manifestations and a classical mutation, highlighting the possible occurrence of spinal ischemic manifestations in FD. However, because FD patients are not regularly subjected to spinal MRI, spinal lesions might be underestimated in FD (5).

Spinal cord infarctions and infarctions in the posterior circulation are predominantly of cardio-embolic origin. Extensive stroke work-up, including long-term ECG and transesophageal echocardiography, was inconspicuous. Although we could not detect any suspicious cardiac findings indicating a potential cardio-embolic mechanism (e.g. atrial fibrillation), this mechanism appears likely in our case. Reviewing the literature, FD patients with cardiac involvement often have brain infarctions with embolic pattern. In summary, our patient experienced two infarctions, probably caused by recurrent cardiac emboli.

The CSF findings in our patient are in line with previous reports, describing an “aseptic meningitis” or “chronic meningitis” with mild to moderate pleocytosis and elevated total protein levels suggestive of a disturbed blood-cerebrospinal fluid barrier (9). The additional cerebral ischemic strokes in this patient support the suggested vascular etiology and might underline that ischemia of the central nervous system in FD is not limited to the cerebrum.

In conclusion, ischemic lesions of the spinal cord can occur in patients with FD and need to be investigated. The diagnosis of FD should not be prematurely excluded in the presence of spinal lesions. Most importantly, since appropriate disease-modifying therapies are available for both diseases, the ability to distinguish FD from MS remains essential. Therefore, we suggest deleting the red flag for the presence/absence of spinal lesions in the differential diagnosis between MS and FD.

## Patient Perspective

Since initially a diagnosis could not be established, I felt very helpless at that time. In the end, I hope that physicians all over the world have learned something from reading my case.

Fabry disease was biochemically diagnosed seven years before occurrence of the complete spinal cord syndrome. Infectious disorders were excluded as possible causes in serum and CSF. The antibody screening in serum was negative.

## Data Availability Statement

The original contributions presented in the study are included in the article/supplementary material. Further inquiries can be directed to the corresponding author.

## Ethics Statement

Written informed consent was obtained from the individual(s) for the publication of any potentially identifiable images or data included in this article.

## Author Contributions

JK, FG, and TD conceived the study and defined the concept. JK, FG, TD, HW, EB, CP, MH, SM, and ML collected and interpreted the data. JK wrote the initial draft of the manuscript. All authors contributed to the article and approved the submitted version. All authors agreed to be accountable for all aspects of the work by ensuring that questions related to the accuracy or integrity of any part of the work are appropriately investigated and resolved.

## Conflict of Interest

JK: received honoraria for lecturing from Biogen, Novartis, Genzyme, Merck, Mylan, and Teva and financial research support from Sanofi Genzyme and Novartis. EB: received research grants and speaker honoraria from Sanofi Genzyme, Shire/Takeda, and Amicus Therapeutics. ML: received speaker honoraria from Amicus Therapeutics, Sanofi Genzyme, and Shire/Takeda and advisory board honoraria from Sanofi Genzyme. HW: received compensation for serving on Scientific Advisory Boards/Steering Committees for Bayer Healthcare, Biogen Idec, Sanofi Genzyme, Merck Serono, and Novartis. He has received speaker honoraria and travel support from Bayer Vital GmbH, Bayer Schering AG, Biogen, CSL Behring, EMD Serono, Fresenius Medical Care, Genzyme, Merck Serono, Omniamed, Novartis, and Sanofi Aventis. He has received compensation as a consultant from Biogen Idec, Merck Serono, Novartis, Roche, and Sanofi-Genzyme. HW also received research support from Bayer Healthcare, Bayer Vital, Biogen Idec, Merck Serono, Novartis, Sanofi Genzyme, Sanofi US, and Teva. SM: received honoraria for lecturing and travel expenses for attending meetings from Almirall, Amicus Therapeutics Germany, Bayer Health Care, Biogen, Celgene, Diamed, Genzyme, MedDay Pharmaceuticals, Merck Serono, Novartis, Novo Nordisk, ONO Pharma, Roche, Sanofi-Aventis, Chugai Pharma, QuintilesIMS, and Teva. His research is funded by the German Ministry for Education and Research (BMBF), Deutsche Forschungsgemeinschaft (DFG), Else Kröner Fresenius Foundation, German Academic Exchange Service, Hertie Foundation, Interdisciplinary Center for Clinical Studies (IZKF) Muenster, German Foundation Neurology, and by Almirall, Amicus Therapeutics Germany, Biogen, Diamed, Fresenius Medical Care, Genzyme, Merck Serono, Novartis, ONO Pharma, Roche, and Teva. TD: received honoraria and travel expenses from Genzyme, Shire, Bristol-Myers Squibb, Boehringer-Ingelheim Pharma, Sanofi Aventis, Wisai, Novartis, Bayer Vital, Merz Pharma, Actelion, and Lundbeck for serving as a speaker and consultant. He received research support from Genzyme, Shire, and Actelion and grants from Novartis and Merz Pharma for conducting dementia studies.

The remaining authors declare that the research was conducted in the absence of any commercial or financial relationships that could be construed as a potential conflict of interest.

## Abbreviations

FD, Fabry disease; FLAIR, fluid-attenuated inversion recovery; MS, multiple sclerosis; WML, white matter lesion.
